# COVID-19 trials: declarations of data sharing intentions at trial registration and at publication

**DOI:** 10.1186/s13063-021-05104-z

**Published:** 2021-02-18

**Authors:** Rebecca Li, Megan von Isenburg, Marcia Levenstein, Stan Neumann, Julie Wood, Ida Sim

**Affiliations:** 1 Vivli, Cambridge, USA; 2 Harvard, Center for Bioethics, Boston, MA USA; 3 Duke University, Durham, NC USA; 4 University of California, San Francisco, CA USA

## Abstract

**Background:**

The sharing of individual participant-level data from COVID-19 trials would allow re-use and secondary analysis that can help accelerate the identification of effective treatments. The sharing of trial data is not the norm, but the unprecedented pandemic caused by SARS-CoV-2 may serve as an impetus for greater data sharing. We sought to assess the data sharing intentions of interventional COVID-19 trials as declared in trial registrations and publications.

**Methods:**

We searched ClinicalTrials.gov and PubMed for COVID-19 interventional trials. We analyzed responses to ClinicalTrials.gov fields regarding intent to share individual participant level data and analyzed the data sharing statements in eligible publications.

**Results:**

Nine hundred twenty-four trial registrations were analyzed. 15.7% were willing to share, of which 38.6% were willing to share immediately upon publication of results. 47.6% declared they were not willing to share. Twenty-eight publications were analyzed representing 26 unique COVID-19 trials. Only seven publications contained data sharing statements; six indicated a willingness to share data whereas one indicated that data was not available for sharing.

**Conclusions:**

At a time of pressing need for researchers to work together to combat a global pandemic, intent to share individual participant-level data from COVID-19 interventional trials is limited.

Clinical trials are the best source of evidence for guiding the treatment of COVID-19, the disease caused by the novel coronavirus SARS-CoV-2. In this time of global pandemic, it is imperative that results from COVID-19 treatment trials be shared as quickly as possible, ideally with availability of the underlying individual participant-level trial data (IPD) to enable more complex and flexible analyses and data aggregation than is possible with only summary-level results. Such IPD sharing would maximize the clinical and scientific value of the contributions of trial participants and would help accelerate findings to identify effective COVID-19 treatments.

Whereas there are global requirements for trial registration and requirements for summary-level results reporting in the USA [1], IPD sharing is not uniformly required. Some 50 funders worldwide—predominantly in Europe and the UK—require IPD sharing of their fundees [2], but the most wide-reaching IPD sharing policy is from the International Committee of Medical Journal Editors (ICMJE) [3]. The ICMJE’s editorial policies are highly influential in medical publishing despite having only 12 journals as members. In 2017, the ICMJE enacted a key requirement that authors submitting clinical trial reports to these journals must include a statement detailing their IPD sharing plans. They additionally required as of January 2019 that trials must include an IPD data sharing statement in their original trial registration (e.g., on ClinicalTrials.gov) in order for their results to be considered for ICMJE publication. Best practices for sharing IPD have been collated as well for publicly funded trials [4]. If ever there was a time for trialists to commit to sharing participant-level data, this global pandemic is it. We sought to determine the data sharing intents of COVID-19 trials to assess what is arguably the current upper bound of trialist interest in IPD sharing.

## Methods

We analyzed data sharing intentions as declared in two separate datasets: ClinicalTrials.gov registrations of COVID-19 trials and publications of COVID-19 trials.

For COVID-19 trial registrations, we searched ClinicalTrials.gov for interventional trials registered before June 30, 2020, with “COVID-19” or related terms (e.g., SARS-CoV-2, severe acute respiratory syndrome, coronavirus 2, coronavirus 2, 2019-nCoV, 2019 novel coronavirus, Wuhan coronavirus) in the condition field or in the title. For included trials, we downloaded and analyzed these data fields: Overall Status, IPD Data Sharing, IPD Data Sharing Description, and IPD Data Sharing Timeframe. For the “IPD Data Sharing” field, we collated the “Yes,” “No,” and “Undecided” responses. If the response was blank, we classified the response as “No response/Missing.” The IPD Data Sharing Description field contains free-form text explaining “No” and “Undecided” responses in the IPD Data Sharing field. We reviewed these explanations for mismatches on intent to share. For example, if the IPD Data Sharing response was “No” yet the IPD Data Sharing Description field said “the data will be shared upon publication,” the trial was re-classified as a “Yes” on intent to share. Responses to the IPD Data Sharing Timeframe field, which is a free-form text field on when data will be shared, were classified as immediately upon publication, 1 to < 6 months, 6 to 12 months, > 12–24 months, and over 24 months.

For COVID-19 trials publications*,* we searched PubMed in May 2020 using a combination of subject headings and keywords for COVID-19 and clinical trials. No limits on language or date were used, and both protocol design and results reporting publications were eligible. Two independent reviewers (RL and ML) screened the publications against predetermined inclusion criteria for COVID-19-related interventional trials in humans. Differences were adjudicated by a third independent reviewer (IS). We focused our analysis on the data sharing statements, if any, in the publications. For each unique trial in the included publications, we also cross-checked for any corresponding trial registrations and checked the data sharing responses for whether they matched. For both registrations and publications, we additionally detailed their study phases. Patients and the public were not involved in this study.

## Results

### COVID-19 trial registrations

Nine hundred twenty-four trials met our inclusion criteria and were analyzed. 47.5% (439) were pre-recruitment and 48.4% (447) were in the midst of recruiting. The remainder (4.1%) were Completed, Terminated, Withdrawn, or Suspended. As shown in Table 1, 15.7% (145) of the trials were affirmative in their intent to share data answering “Yes” in the IPD Data Sharing field, 14.2% (131) answered “Undecided,” 47.6% (440) answered “No,” and 22.5% (208) were missing a response. Analysis of the field “IPD Data Sharing Timeframe” revealed responses ranging from sharing immediately to 24 months after publication. For the 145 trials responding “Yes” in the IPD Data Sharing field, 38.6% declared the intention to share immediately, 9.6% between 1 and 6 months, 15.1% between 6 and 12 months, and 11% 12 or more months after publication. 25.5% did not specify a timeframe when data would be shared (Table 2).

**Table 1:**
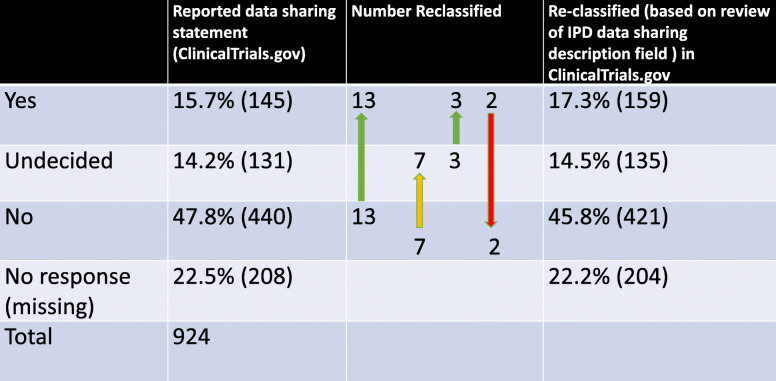
Data sharing statements from ClinicalTrials.gov registrations

**Table 2:** Timing of intended data sharing

Reported timing of initial sharing	% that agreed to share in timeframe
Immediately	56 (38.6%)
1 to < 6 months	14 (9.6%)
6–12 months	22 (15.1%)
12–24 months	16 (11.0%)
No timing given	37 (25.5%)
Total number	145

We compared responses of the IPD Data Sharing and IPD Data Sharing Description fields to check for fidelity and to reclassify the declared intent if appropriate (Table 1). Of the 439 trials that answered “No” to IPD Data Sharing, 50 provided IPD Data Sharing Description responses that confirmed their intent to not share, with two responses specifically citing regulatory rationale. Another 13 of these “No”-sharing trials had data sharing descriptions that actually indicated a willingness to share (e.g., “requests for IPD may be submitted to the PI for review”; “anonymised results will be published in a scientific journal...and posted on the…website”, and “the datasets used and analyzed during the current study will be available from the corresponding author on reasonable request.”). An additional 7 “No”-sharing trials were reclassified as “Undecided” based on their free-text explanations. For trials that answered “Undecided” to IPD Data Sharing, 3 were reclassified as “Yes” based on the explanations in IPD Data Sharing Description. For trials that answered “Yes” to IPD Data Sharing, we reclassified two entries to “No” as they described only sharing between members of the study team rather than broader IPD data sharing. Thus, following this review, the number of Yes’s increased by 14 (145 to 159), the number of Undecideds increased by 4 (131 to 135), and the number of No’s decreased by 19 (440 to 421). Overall, the number of trials that intended to share data changed from 15.7% to 17.2% after reclassification.

### COVID-19 publications

Twenty-eight publications met our criteria, representing 26 unique trials (2 trials spawned 2 manuscripts each). Ten publications described study protocols while the rest were reports of results. The vast majority of the publications (80.8%; 21 of 26) did not include a data sharing statement. The 7 available data sharing statements from 7 unique trials showed that 6 trials (21.4% of 26) were willing to share and 1 trial was unwilling to share their data (Table 3).

**Table 3:** Data sharing statements from publications

	Reported
Yes	21.4% (6)
Undecided	0% (0)
No	3.5% (1)
No response (missing)	75% (21)

Twelve of the 26 published trials had corresponding ClinicalTrials.gov registrations, while 14 trials were registered elsewhere (e.g., EU Clinical Trial Register, the Chinese Clinical trial registry). The 12 trials that were registered in ClinicalTrials.gov accounted for 14 of the 26 publications. Of these, only seven contained data sharing statements. Comparing the data sharing intent in the trial registration versus in the publication, we found 8 cases of discrepancies out of the 12 unique trials (note there were 8 cases as one of the trials that originally declared “yes” to sharing in ClinicalTrials.gov was missing a data sharing statement in the manuscript). Four trials changed from “No” or “Undecided” at registration to clear willingness to share in the publication statement. Two rescinded their intent to share data, and 2 changed from “Undecided” to “Missing.”

## Discussion

Sharing participant-level trial data is intended to enable researchers to build on existing knowledge, ensure reproducibility, reduce duplication, pool data for meta- and other aggregate analysis, and generally accelerate the pace of science. We characterized the current data sharing landscape for COVID-19 interventional trials during this global pandemic and public health emergency as the imperative to share data could not be greater. The addition of data sharing statements in ICMJE journals and ClinicalTrials.gov facilitated this analysis of data sharing practices.

Prior general surveys of data sharing intent (not focused on COVID-19) [5, 6] showed a willingness to share IPD in the 5.5–10.8% range. Thus, our finding that 15.7% of COVID-19 trials are willing to share is a notable increase, but is still dismayingly low when the circumstances are such that trialist desire to share should be highest. Moreover, only 6.1% of trials overall (56/919) committed to sharing data immediately upon publication, and 10% (92/919) within a year of publication. This timeframe may be expeditious in normal times but is alarming given that we are in the midst of a global pandemic where the doubling time of the number of COVID cases can be measured in days and weeks. Reasons for the meager intent to share may include practical challenges such as cost, need for anonymizing data, the challenge of managing data requests, and lack of academic credit for sharing [7–9]. We surmise that trialists’ low rates of sharing may be due more to these practical challenges than to an outright objection to sharing. Twenty-four of the 28 publications were freely available and accessible in PubMed Central, indicating an interest in promoting open science in response to this public health emergency, whether due to socialization or to funder mandates to share full-text articles. Clearly, there is tremendous technical, policy, and cultural work to be done to make IPD sharing more widespread, routine, and timely.

An area of immediate opportunity for improvement is clear and consistent declarations of data sharing intent. We found discrepant data sharing statements within 2.7% of analyzed trial registration records in ClinicalTrials.gov, and the few analyzed publications that had data sharing statements with the exception of one, conflicted in non-trivial ways with the original declared intent at the time of trial registration. These inconsistencies are confusing and complicate efforts to tally or enforce IPD sharing. Researchers should be provided with education and support for accurately completing trial registration fields and should be encouraged to provide data sharing statements with all publications to reaffirm their data sharing commitments.

This study has the following limitations. First, we analyzed only ClinicalTrials.gov and not EU Clinical Trial Register or registers of the World Health Organization’s International Clinical Trials Registry Platform. However, ClinicalTrials.gov contains the majority of the world’s trial registrations. A second limitation is that we only searched PubMed and not additional databases; however, this is accepted practice in many COVID-19-related rapid reviews. Third, most of the publications we analyzed were not from ICMJE journals and were thus not required to include data sharing statements. Finally, this analysis is a snapshot of trial registrations and publications from the early days of the SARS-C0V-2 pandemic and not intended to be comprehensive. Data sharing intent and actual data sharing behavior may evolve as science responds to this challenging pandemic.

In conclusion, we found that fewer than one out of six COVID-19 interventional clinical trials have committed to sharing their participant-level data. Of these, 63% are committed to sharing within 12 months of publication. At a time of pressing need for researchers to work together to combat a global pandemic, data sharing intent is limited and may restrict the scientific value of COVID-19 trials.

## Data Availability

Following publication, all primary data tables will be upon request to rli@vivli.org.
